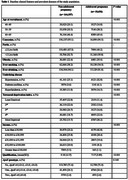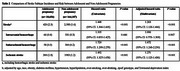# Oral Concurrent Session 8 – Hypertension: Friday, February 13, 2026 8:00 AM – 10:00 AM

**DOI:** 10.1002/pmf2.70153

**Published:** 2026-02-09

**8:00 AM – 8:15 AM**

## 82 | Prospective Evaluation of Platelet Activity and Aspirin Responsiveness in Pregnancy

Christina A. Penfield^1^; Ariel Schapp^2^; Elliot Luttrell‐Williams^3^; Anais Hausvater^3^; Andre A. Robinson^1^; Yuhe Xia^4^; Matthew Muller^3^; Madison S. House^5^; Valeryia Avtushka^1^; Lauren Kennedy^3^; Rafid Quadir^3^; Justin S. Brandt^6^; Gwendolyn P. Quinn^7^; Ashley S. Roman^6^; Dana R. Gossett^7^; Jeffrey S. Berger^3^

^1^Division of Maternal‐Fetal Medicine, Department of Obstetrics and Gynecology, NYU Grossman School of Medicine, New York City, NY; ^2^Division of Hematology Department of Medicine, NYU Grossman School of Medicine, New York City, NY; ^3^Leon H. Charney Division of Cardiology, Department of Medicine, NYU Grossman School of Medicine, New York City, NY; ^4^Division of Biostatistics, NYU Grossman School of Medicine, New York City, NY; ^5^NYU Grossman School of Medicine, New York City, NY; ^6^Division of Maternal‐Fetal Medicine, Department of Obstetrics and Gynecology, NYU Grossman School of Medicine, New York, NY; ^7^Department of Obstetrics and Gynecology, NYU Grossman School of Medicine, New York City, NY

**Objective**: Platelet activity may not be adequately inhibited by low dose aspirin (ASA) in some people at risk for hypertensive disorders of pregnancy (HDP), limiting the potential benefits of ASA prophylaxis. We investigated platelet activity in pregnancy and its association with ASA responsiveness.

**Study Design: P**regnancy **O**utcomes and **P**latelet **P**henot**Y**pes (POPPY) was a prospective cohort recruited in the first trimester and classified as high or low risk for HDP based on US Preventative Task Force Criteria. The high‐risk group was recommended ASA 81mg daily (ASA group) and the low‐risk group was not (controls). We evaluated serum thromboxane B2 (TxB2) and platelet indices at 11–14 weeks (pre‐ASA) and 24–28 weeks (on ASA). Platelet aggregation was evaluated with arachidonic acid (AA) +/− in vitro ASA, submaximal agonists (adenosine diphosphate [ADP], collagen, epinephrine [EPI]), and spontaneous platelet aggregation (SPA). ASA non‐response was defined as >20% platelet aggregation with high‐dose AA at 24–28 weeks.

**Results**: Among 66 participants, 56 completed both blood draws (41 ASA compliant and 15 controls; Table). Among controls, platelet count and size did not change, whereas platelet immaturity and platelet aggregation increased with submaximal agonist stimulation. In the ASA group, ASA induced a 99% decrease in serum TxB2 (26.3 to 0.3 ng/ml, *p* < 0.001) and 70% decrease in platelet aggregation with AA (89% to 15%, *p* < 0.001). Submaximal agonists elicited a heterogeneous response; with 29%, 53%, and 31% decrease in platelet aggregation with ADP, collagen, and EPI, respectively (*p* < 0.05 for each). Despite near complete TxB2 inhibition, 31% of high‐risk participants were ASA non‐responders. ASA non‐responders had significantly higher levels of SPA and platelet aggregation with submaximal agonists at baseline (Figure).

**Conclusion**: Platelet immaturity and aggregation increase during pregnancy. While ASA reduced platelet aggregation, nearly 1 in 3 were ASA non‐responsive, suggestive of inadequate platelet inhibition. First trimester platelet activity may identify non‐responders with potential clinical implications.

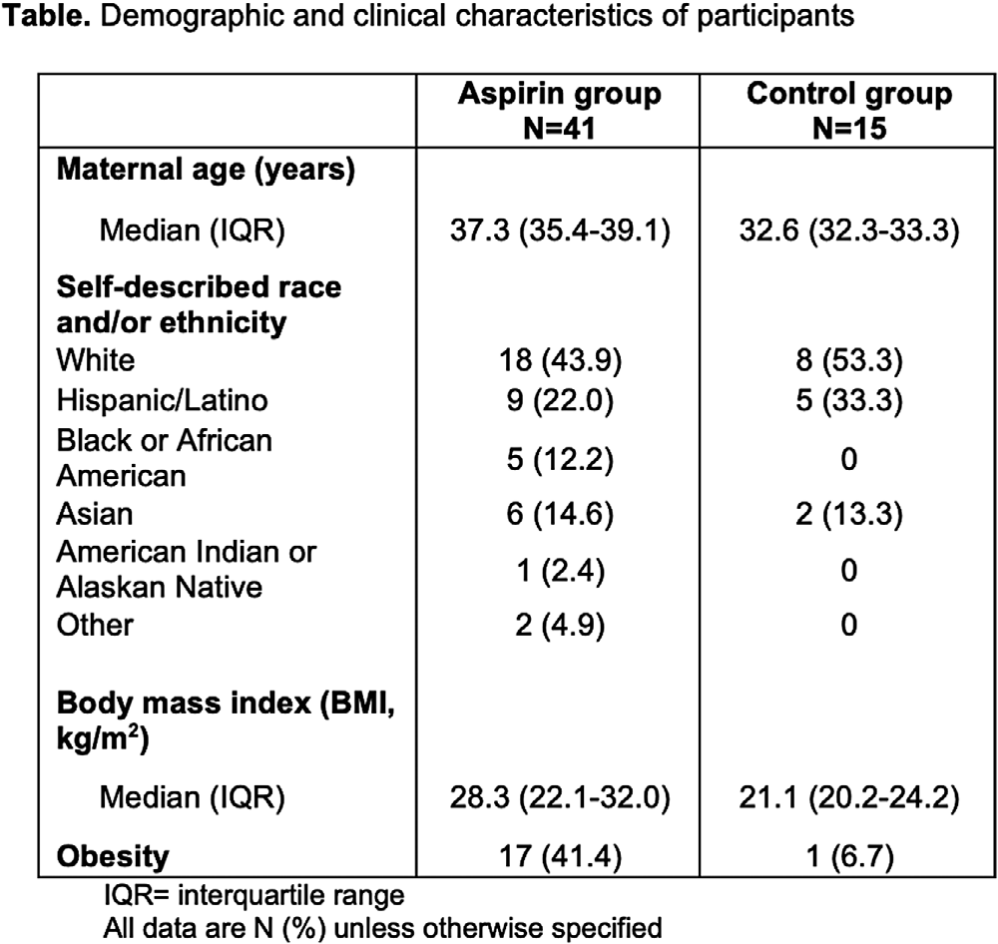

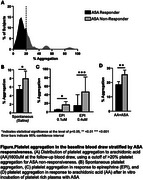

**8:15 AM – 8:30 AM**

## 83 | **Association of First Trimester Glycemia With Hypertensive Disorders of Pregnancy**

Lynn M. Yee^1^; Nicola Lancki^2^; Patrick M. Catalano^3^; Francesca Facco^4^; Maisa Feghali^5^; William A. Grobman^6^; Erin S. LeBlanc^7^; William L. Lowe^8^; Audrey Merriam^9^; Mirella Mourad^10^; Caryn E.S. Oshiro^11^; Camille E. Powe^12^; Uma M. Reddy^13^; Jennifer Sherr^14^; Alexandra C. Spadola^15^; Kimberly K. Vesco^7^; Erika F. Werner^15^; Noelia Zork^13^; Denise Scholtens^1^; Dwight J. Rouse^16^

^1^Northwestern University Feinberg School of Medicine, Chicago, IL; ^2^Northwestern University, Chicago, IL; ^3^MGH REU, Boston, MA; ^4^UPMC, UPMC, PA; ^5^Magee Women's Research Institute, University of Pittsburgh, Pittsburgh, PA; ^6^Warren Alpert Medical School of Brown University, Providence, RI; ^7^Kaiser Permanente Center for Health Research, Portland, OR; ^8^Northwestern University, Northwestern University, IL; ^9^Yale School of Medicine, Yale School of Medicine, CT; ^10^Division of Maternal‐Fetal Medicine, Department of Obstetrics and Gynecology, Columbia University Irving Medical Center, New York, NY; ^11^Kaiser Permanente Hawaii, Center for Integrated Health Care Research, Honolulu, HI; ^12^Massachusetts General Hospital, Boston, MA; ^13^Columbia University Irving Medical Center, New York, NY; ^14^Yale School of Medicine, New Haven, CT; ^15^Tufts University School of Medicine, Boston, MA; ^16^Women & Infants Hospital of Rhode Island / Warren Alpert Medical School of Brown University, Providence, RI

**Objective**: Nocturnal hyperglycemia in non‐pregnant adults is associated with the development of hypertension. Pregestational diabetes mellitus (DM) is a risk factor for hypertensive disorders of pregnancy (HDP) but, in the absence of DM, the relationship between first trimester glucose values and HDP is not known. We therefore evaluated the association of first trimester glycemia with HDP.

**Study Design**: Planned analysis of data from a US multisite, prospective cohort study of gravidas without pregestational DM who completed a 2‐hour 75 g oral glucose tolerance test (OGTT) and then wore a continuous glucose monitor (CGM) for ≥5 days at 10–14 wks. OGTT and CGM thresholds for this analysis were chosen because they optimized prediction of gestational DM; the CGM threshold was a dichotomous measure of nocturnal (12 am–6 am) glucose >133 mg/dL for ≥9% of values. We also evaluated mean nocturnal glucose and percent nocturnal time >133 mg/dL. HDP was abstracted by trained staff using standard criteria. Sensitivity analyses excluded gravidas with chronic hypertension (cHTN). Poisson regression models with robust error variance were used to estimate relative risk (RR) and 95% confidence intervals (CI) for HDP adjusted for confounders defined a priori; a second model further adjusted for aspirin use.

**Results**: Of 2178 gravidas enrolled from 2021–24, 2072 (95%) had OGTT data and 1930 (89%) had CGM data available for this analysis. Participant characteristics generally reflected the US population: 45% nulliparous, 31% publicly insured, 62% overweight/obese, 6% cHTN, and 35% aspirin use. Overall, 17.5% developed HDP (Figure). The 10–14 wk OGTT values, whether evaluated dichotomously or continuously, were not associated with HDP (Table). In contrast, the prespecified CGM threshold was associated with HDP (aRR 1.32, 95% CI 1.05–1.65) as was percent nocturnal time >133 mg/dL (Table). Findings were similar after adjusting for aspirin use (aRR 1.30, 95% CI 1.04–1.62) and excluding gravidas with cHTN.

**Conclusion**: First trimester nocturnal glycemia measured by CGM, but not glycemia measured by OGTT, was associated with HDP.

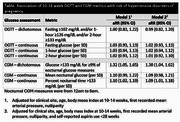

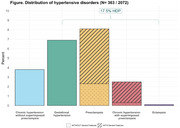

**8:30 AM – 8:45 AM**

## 84 | **Multimodal Health System Intervention for Postpartum Hypertension: Influence on Readmission Rates**

Claire R. Conklin^1^; Karen L. Li^2^; Jane W. Liang^3^; Monique Hedderson^4^; Susanna D. Mitro^5^; Mara B. Greenberg^6^

^1^Kaiser Permenente, Kaiser/san Francisco, CA; ^2^Kaiser Permenente, Kaiser/northern california, CA; ^3^Kaiser Permenente, Kaiser/norther California, CA; ^4^Kaiser Permenente, Kasier/Northern California, CA; ^5^Kaiser Permenente, Kaiser/Norther California, CA; ^6^Kaiser Permenente, Kaiser/Oakland, CA

**Objective**: Hypertensive disorders of pregnancy (HDP) increase risk of postpartum (PP) readmission compared to non‐HDP pregnancies. Investigation of safely reducing HDP‐associated PP readmission is critical. We studied readmission rates associated with a system‐wide initiative in Kaiser Permanente Northern California (KPNC) to standardize PP HDP management.

**Study Design**: Retrospective cohort study of all pregnancies with HDP and a live birth delivered 2019–2023 in KPNC using EHR data. We analyzed patient characteristics and outcomes related to a 2022 multimodal intervention which included implementing delivery discharge BP goals, increasing nifedipine use as a single agent anti‐hypertensive, and enrollment in Regional Perinatal Service Center (RPSC) telehealth PP BP monitoring program. We used interrupted time series (ITS) analyses to examine monthly outcomes (readmission and ED visits), process measures, and severe maternal morbidity (SMM) across 3 time periods: pre‐intervention (T1, 1/1/2019–12/31/2021), intervention (T2, 1/1/2022–9/30/2022) and post‐intervention (T3, 10/1/2022–12/31/2023).

**Results**: We included 54,607 pregnancies with HDP. Patients were mostly diagnosed with gestational hypertension (45%), non‐White race (55%), BMI ≥30 (44%), nulliparity (50%), and commercially insured (87%). Unadjusted proportions of PP readmission were unchanged in T1, T2 and T3 (4.2%, 4.2%, 4.5%, Table 1, Figure 1) while proportions of ED visits increased and SMM decreased slightly (Table 1, Figure 1). ITS analysis showed no significant trend changes over time in readmission or SMM, despite an upward trend in ED visit rate in T3 vs T1 (T3 monthly change in slope 1.91 per 1000 (95% CI 0.43‐3.40, *p* = 0.01)).  Nifedipine use and RPSC enrollments increased (Table 1, Figure 1), however ITS analysis did not identify significant change in the rate in T3 compared to T1.

**Conclusion**: Despite large changes in antihypertensive medication prescribing practices and home BP monitoring enrollments over time, and an uptrend in ED visits, PP readmission rates remained unchanged among patients with HDP.

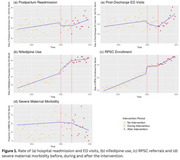

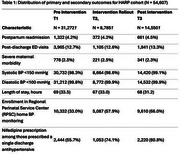

**8:45 AM – 9:00 AM**

## 85 | **CATAlytic Antagonist of PolyCOMB (CATACOMB) as a Master Regulator of the Placental Epigenome in Preeclampsia**

Sydney E. Dautel^1^; Guomao Zhao^1^; Meera M. Thakkar^1^; Sunayana Srinivasan^1^; William E. Ackerman, IV^2^; Catalin S. Buhimschi^1^; Andrea Piunti^3^; Irina A. Buhimschi^2^

^1^University of Illinois Chicago, University of Illinois Chicago, IL; ^2^University of Illinois Chicago, Chicago, IL; ^3^University of Chicago, University of Chicago, IL

**Objective**: Epigenetic mechanisms regulate placental invasion, a process disrupted in PE. DNA methyltransferases (DNMT) and Polycomb Repressive Complex 2 (PRC2) are epigenetic gene silencers via different yet complementary mechanisms. While DNMTs methylate DNA, PRC2 is responsible for methylating histone H3 (H3K27me3). The newly described molecule CATACOMB functions as a PRC2 inhibitor. Due to their key roles in cellular proliferation/differentiation PRC2 and CATACOMB are considered oncotherapeutic agents. Herein we investigated the placental expression of PRC2, CATACOMB and their relationship to DNMTs in early onset PE (<34 w).

**Study Design**: Using DESeq2, we interrogated an RNA sequencing (RNAseq) dataset of placental villous tissue (GSE114691) from pregnancies with (*n* = 20) and without PE (*n* = 21) for expression of PRC2 subunits (EZH1, EZH2 and SUZ12), CATACOMB, and DNMT1, 3A, and 3B. Immuno‐histochemistry (IHC) for EZH2, SUZ12, CATACOMB and their methylation product H3K27me3 was sought in PE (*n* = 3) and non‐PE placenta across gestational age (*n* = 14: 1^st^ trim: *n* = 4; 2^nd^ trim, *n* = 6; 3^rd^ trim *n* = 4).

**Results**: PRC2 subunits and CATACOMB were expressed in the human placenta and distinctively correlated with DNMTs. CATACOMB was positively correlated with EZH1 (*r* = 0.4, *p* < 0.05), while it correlated negatively with EZH2 (*r* = −0.4, *p* < 0.05) and DNMT1(*r* = −0.3, *p* < 0.05). EZH1 mRNA expression increased across gestation (*r* = 0.7, *p* < 0.001) as EZH2 decreased (*r* = −0.8, *p* < 0.001). PE increased expression of EZH1 (*p* < .01) and CATACOMB (*p* < 0.001) yet decreased EZH2 (*p* < 0.001) supporting the opposing regulation among different epigenetic regulators (Figure). IHC localized CATACOMB, EZH2 and H3K27me3 to syncytial sprouts of the 1^st ^and 2^nd^ trim placentas and syncytiotrophoblast of placenta affected by PE.

**Conclusion**: We discovered CATACOMB, PRC2 subunits and it's end‐product (H3K27me3) are differentially expressed in the human placenta, in a distinct pattern compared to DNMTs. The conspicuous expression of syncytial sprouts in early gestation and in PE suggest the PRC2/CATACOMB complex may play key roles in regulating trophoblast invasion.

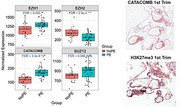

**9:00 AM – 9:15 AM**

## 86 | **Using Machine Learning to Predict Pre‐Eclampsia Before 20 Weeks Gestation**

Lauren M. Kucirka^1^; Mohammad Kibria^2^; Leyu Dai^2^; Safoora Masoumi^2^; Mian Wei^3^; Metin Gurcan^4^; David Page^3^; Alison M. Stuebe^5^

^1^UNC‐Chapel Hill, Chapel Hill, NC; ^2^University of North Carolina, Chapel Hill, NC; ^3^Duke University, Durham, NC; ^4^Wake Forest University, Winston‐Salem, NC; ^5^University of North Carolina at Chapel Hill School of Medicine, Chapel Hill, NC

**Objective**: Early identification of patients at risk of pre‐eclampsia is critical to deploy protective interventions such as low dose aspirin and home blood pressure monitoring. Our study aimed to 1) develop a machine learning‐based pre‐eclampsia prediction model using data available ≤20 weeks gestation, 2) compare model performance to use of ACOG aspirin criteria alone for identifying patients who will develop pre‐eclampsia, and 3) identify novel pre‐eclampsia risk factors.

**Study Design**: Patients who delivered at a large academic medical system between 2016 and 2023 and had at least 1 visit prior to 20 weeks were included. We used random forest machine learning (ML) algorithms to predict pre‐eclampsia using laboratory, medication, vital sign, diagnosis, social determinants of health,  and demographic data captured in the electronic medical record (EMR) up to 20 weeks gestation. The area under the curve (AUC) was calculated to assess model performance. An information gain algorithm was applied to select the top 50 of >1300 variables. We then determined which patients met ACOG aspirin criteria. The sensitivity and specificity of ACOG guidelines versus our ML model to predict future pre‐eclampsia were calculated and compared using 20% of the sample withheld for testing.

**Results**: In total 24,026 patients met inclusion criteria; 6% were ultimately diagnosed with pre‐eclampsia. Our ML model had an AUC of 0.79. Diastolic blood pressure, presence of cerebrovascular disorder, and pulse pressure were identified as top predictors of subsequent pre‐eclampsia that are not currently included in ACOG criteria (Figure). Compared to ACOG criteria, our ML model had higher sensitivity (91% vs 86%) at equal specificity (36%).

**Conclusion**: Our model had higher sensitivity compared to use of ACOG guidelines alone. Cerebrovascular disorders, early diastolic BP and pulse pressures emerged as  important predictors; adding this information to ACOG criteria may improve early identification of those at highest risk of pre‐eclampsia.

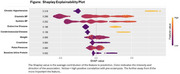

**9:15 AM – 9:30 AM**

## 87 | **A Randomized Controlled Trial for Postpartum Preeclampsia Antihypertensive Treatment (PPAT)**

Nadine Sunji^1^; Amy Y. Pan^1^; Zaira Peterson^1^; Eleanor Saffian^1^; Alyssa M. Hernandez^1^; Anna Palatnik^2^

^1^Medical College of Wisconsin, Medical College of Wisconsin, WI; ^2^Medical College of Wisconsin, MILWAUKEE, WI

**Objective**: To assess whether tight postpartum blood pressure (BP) control in patients with gestational hypertension (GHTN) and preeclampsia reduces hospital readmissions, healthcare utilization, and elevated BP within 6 weeks postpartum compared with standard of care.

**Study Design**: This single‐center randomized controlled trial (June 2020‐June 2025, with a 1.5‐year COVID‐19 hiatus) enrolled postpartum (day 0–4) patients aged ≥18 years with GHTN or preeclampsia and at least two postpartum BP ≥140/90 mmHg. Before discharge, participants were randomized to either antihypertensive treatment (nifedipine XL 30 mg daily or labetalol 200 mg BID) or standard of care (medication at provider discretion). All participants received home BP monitoring. The primary outcome was hospital readmission for hypertension within 6 weeks postpartum. Secondary outcomes included unplanned healthcare utilization for hypertension within 6 weeks postpartum, severe hypertension (≥160/110 mmHg) at 1‐2 weeks postpartum, stage I (≥130/80 mmHg) and II (≥140/90 mmHg) hypertension at 6 weeks postpartum, and mean BP at 1–2 and 6 weeks postpartum. An intention‐to‐treat analysis was performed.

**Results**: A total of 188 patients were randomized (*n* = 94 per group) with comparable baseline characteristics (Table 1). Postpartum readmissions were lower in the treatment group (8.5%) compared to controls (17.0%), though not statistically significant (*p* =  .080; Table 2). Rates of unplanned healthcare utilization and unplanned clinic visits were also lower in the treatment group but did not reach statistical significance. However, at 1–2 weeks postpartum, severe hypertension was significantly less common in the treatment group (9.4% vs. 20.9%, *p* = 0.036), and mean systolic BP was significantly lower (137.4 vs. 143.1 mmHg, *p* =  .047). By 6 weeks postpartum, mean BP and hypertension rates were similar between groups.

**Conclusion**: Tight postpartum BP control in patients with GHTN or preeclampsia reduced mean systolic BP and incidence of severe hypertension at 1–2 weeks postpartum but did not significantly decrease postpartum hospital readmissions.

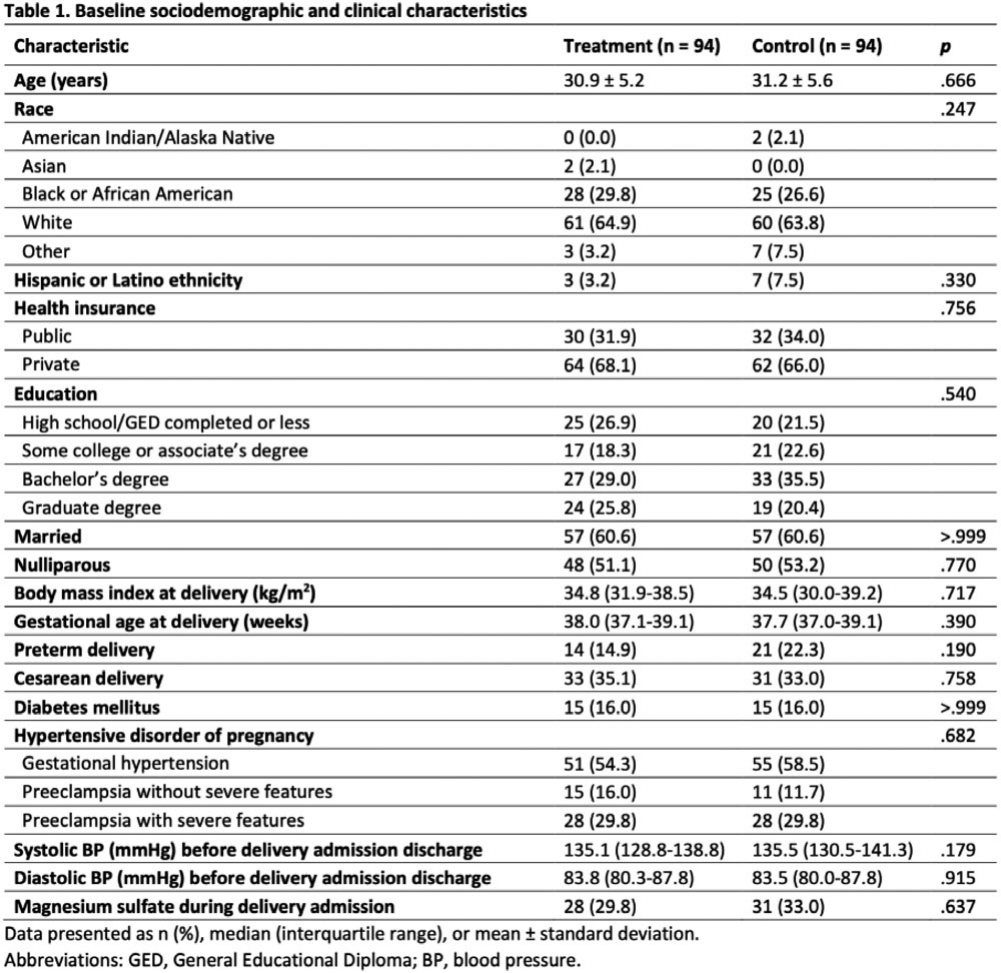

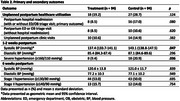

**9:30 AM – 9:45 AM**

## 88 | **Cost‐Effectiveness of Serial Laboratory Monitoring During Expectant Management of Early Preeclampsia With Severe Features**

Zakaria Doughan^1^; Yossi Bart^2^; Joe Haydamous^3^; Wissam Akkary^2^; Ahmed Zaki Moustafa^4^; Baha M. Sibai^2^

^1^Department of Obstetrics and Gynecology, McGovern Medical School at UT Health, Houston, Houston, TX; ^2^Division of Maternal‐Fetal Medicine, Department of Obstetrics, Gynecology and Reproductive Sciences, McGovern Medical School at UTHealth Houston, Houston, TX; ^3^Department of Obstetrics, Gynecology and Reproductive Sciences, McGovern Medical School at UTHealth Houston, Department of Obstetrics and Gynecology, McGovern Medical School at UT Health, Houston, TX; ^4^Division of Maternal‐Fetal Medicine, Department of Obstetrics, Gynecology and Reproductive Sciences, McGovern Medical School at UTHealth Houston, University of Texas ‐ Houston, TX

**Objective**: Current guidelines lack clear recommendations on the optimal frequency of serial lab testing during expectant management of severe preeclampsia. This study aimed to describe the effect of serial laboratory tests on clinical decision‐making and the cost per patient.

**Study Design**: A retrospective study of singletons diagnosed with preeclampsia with severe features between 23w0d and 33w3d (2016–2025), conducted at a single quaternary care center. Patients were excluded if they delivered within one day of admission or had contraindications for expectant management on presentation (eclampsia, HELLP syndrome, stroke, AKI, pulmonary edema, IUFD, and abruption). For each patient, we recorded the number of tests until delivery and associated costs based on institutional pricing. The primary outcome was the proportion of deliveries prompted by abnormal tests. Secondary outcomes included total and average lab‐related cost per patient.

**Results**: A total of 370 patients met inclusion criteria. The median number of tests per patient was 4 (IQR 3–7), with labs drawn on average every 1.6 days (±2.4). The average cost of serial labs per admission was $5,055 (±$3,893), with a median of $4,024 (IQR $2,094–$6,423), totaling $1.87 million over 10 years. Eleven patients (3%) were delivered solely due to abnormal tests; all delivered within 4 days of diagnosis (mean 2.4 ± 1.4 days), and most (6/11) had abnormal labs at presentation that worsened by the time of delivery (Figure 1). Additional 19 patients (5%) had lab abnormalities plus other indications for delivery, most commonly uncontrolled hypertension (11/19), and had longer latency to delivery (mean 7.7 ± 6.6 days; *p* = 0.003).

**Conclusion**: In this cohort of expectantly managed patients with preeclampsia with severe features before 34 weeks, only 3% were delivered solely for abnormal labs, all within 4 days of diagnosis. Most patients with eventual lab abnormalities on serial draws had other concomitant delivery indications. These findings suggest that frequent lab testing may have limited impact on clinical decision‐making and should be weighed against its associated cost.

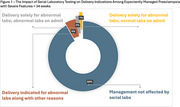

**9:45 AM – 10:00 AM**

## 89 | **Adolescent Pregnancy and Increased Risk of Stroke: A Prospective Population‐Based UK Biobank Study**

Haemin Kim^1^; Mi‐Young Oh^2^; Sang‐Hyuk Jung^3^; Young Mi Jung^4^; Manu Shivakumar^3^; Dokyoon Kim^3^; Ji Hoi Kim^5^; Jeesun Lee^6^; So Hee Kim^5^; Chan‐Wook Park^7^; Joong Shin Park^7^; Seung Mi Lee^8^

^1^Kyungpook National University Chilgok Hospital, Daegu, Taegu‐jikhalsi; ^2^Bucheon Sejong Hospital, Bucheon, Kyonggi‐do; ^3^University of Pennsylvania, Philadelphia, PA; ^4^Department of Obstetrics and Gynecology, Seoul National University Bundang Hospital, Seongnam‐si, Kyonggi‐do; ^5^Seoul National University Hospital, Seoul, Seoul‐t'ukpyolsi; ^6^Gyeongsang National University Hospital, Jinju‐si, Kyongsang‐namdo; ^7^Department of Obstetrics and Gynecology, Seoul National University Hospital, Seoul National University College of Medicine, Seoul, Republic of Korea, Seoul, Seoul‐t'ukpyolsi; ^8^Department of Obstetrics and Gynecology, Seoul National University College of Medicine, Seoul, Seoul‐t'ukpyolsi

**Objective**: Pregnancy‐induced cardiovascular changes during adolescence may affect cerebrovascular development and increase susceptibility to stroke risk later in life. This study aimed to investigate the association between adolescent pregnancy and long‐term risk of stroke, including subtype‐specific risks, in a large prospective cohort.

**Study Design**: The UK Biobank is a prospective cohort study conducted between 2006 and 2010, comprising more than 500,000 participants from the United Kingdom. For this analysis, we included 220,247 women who had reported at least one live birth. Adolescent pregnancy was defined as first pregnancy before age 20. The incidence of new‐onset stroke after enrollment was compared according to the history of adolescent pregnancy using Cox proportional hazards models, adjusting for age, body mass index, diabetes, hypertension, smoking status and alcohol consumption.

**Results**: Of the 220,247 women, 18,656 had a history of adolescent pregnancy. A total of 3550 women (1.6%) experienced new‐onset stroke during follow‐up. Of these, 717 had intracerebral hemorrhage (ICH), 429 had subarachnoid hemorrhage (SAH), and 2718 had ischemic stroke. The risk of stroke was significantly higher in the adolescent pregnancy group compared to the non‐adolescent pregnancy group (adjusted hazard ratio [HR] 1.263, *p* < 0.001). Specifically, the risks of SAH and ischemic stroke were significantly elevated in the adolescent pregnancy group (adjusted HR 1.672, *p* < 0.001 and adjusted HR 1.271, *p* < 0.001, respectively).

**Conclusion**: Adolescent pregnancy is associated with an increased risk of stroke, particularly subarachnoid hemorrhage and ischemic stroke. Further research is needed to understand the underlying mechanisms and clinical implications of adolescent pregnancy for stroke risk assessment.